# Effects of Housing Deprivation on Health: Empirical Evidence from Spain

**DOI:** 10.3390/ijerph20032405

**Published:** 2023-01-29

**Authors:** Demetrio Carmona-Derqui, Jonathan Torres-Tellez, Alberto Montero-Soler

**Affiliations:** 1 Facultad de Economía, Universidad de Granada, 18071 Granada, Spain; 2 Facultad de Economía, Universidad de Málaga, 29071 Málaga, Spain; 3 Facultad de Derecho, Pablo de Olavide University, 41013 Seville, Spain; 4 Department of Public Finance, Economic Policy and Political Economy, Universidad de Málaga, 29071 Málaga, Spain

**Keywords:** housing deprivation, health, multilevel

## Abstract

Severe housing deprivation rates in Spain have tripled in just four years, affecting 3.4% of the population in 2020, with a higher incidence among the low-income population (9.2%). Despite the social aspect of the problem, minimal research has been carried out in Spain on the effects that the various forms of housing deprivation have on health. This study analyzes the impact of housing deprivation on health outcomes, with the objective of achieving results that facilitate the creation of improved public policies. Microdata are used from the Living Conditions Survey carried out by the National Institute of Statistics for the period 2009–2019, and several multilevel logistic regression models are presented to control for possible regional differences. The results show that the elements with the greatest effect on objective health are noise, leaks and harmful temperatures in housing. In addition, environmental factors, such as pollution, neighborhood crime and the number of units in a given apartment building, can be added to the list. As a result, we conclude that there are certain structural and environmental elements in housing and the environment in which is located that have a more intense impact on objective health and on the subjective perception of a person’s state of health.

## 1. Introduction

Housing is a basic human need, where activities within the private sphere of each person are carried out, and the earliest socialization processes begin. Beyond providing the foundation for vital needs such as shelter, security and privacy, it is one of the essential material elements that enable other human rights, such as education and health, to be exercised [1].

The physical and structural characteristics of housing affect people’s quality of life. As such, housing deprivation, which is understood not only as a lack of housing, but also as those situations in which a dwelling’s physical characteristics do not meet the minimum standards, due to the scarcity of economic resources of the affected person [2], undermines people’s well-being, increases the risk of poverty and social exclusion, and affects their health, among other things.

In Europe, the economic crisis of 2008 went far beyond foreclosures. It had a direct impact both on the affordability of housing and on the possibility of improving its physical and structural characteristics. This context was later aggravated by the consequences of the COVID-19 pandemic, when the confinement for several months allowed people to more intensely analyze the deficiencies that their housing had, as shown by early indications from Eurostat for 2020 and 2021 [3,4].

Spain was not spared from this situation, given that it is one of the European Union countries in which access to affordable and adequate housing has become increasingly difficult in recent decades. By 2015, the European head of Eurofound in Spain was already stressing the need to improve accessibility and the general quality of housing in this country [5]. Furthermore, instead of a solution becoming evident in recent years, the situation has worsened. From 2017 to 2020, there was a considerable decline in the physical conditions of Spanish housing, which has caused severe housing deprivation to multiply three-fold in just four years, affecting 3.4% of the population and 9.2% of those in the lowest income bracket [3].

In 2020, according to Eurostat, 19.7% of Spaniards lived in a house with leaks or moisture problems, which represents an increase of 8 percentage points when compared to 2017, and is higher than the European average (13.1%). In addition, according to the National Institute of Statistics [6], 10.8% of the population considered their housing to be too dark, and 14.1% said that there were crime and vandalism problems in their neighborhood.

This situation is worrisome with regard to Spanish health levels, since many studies have shown that housing is an important factor in this context. The groundbreaking work of Chadwick [7] demonstrated that people living in basements had a lower life expectancy than those who had access to more decent housing. Most of the research surrounding this problem has generally focused on how health is affected by the physical and structural conditions of housing and by the environment in which it is located [8]; and experts agree that, generally speaking, the various aspects of housing deprivation have a detrimental effect on the health of those involved [9,10].

This conclusion has been reached after analyzing, for example, how damp conditions, mold, temperature and available space can affect the health of a dwelling’s inhabitants [2]. Various studies have found that overcrowding increases the possibility of infectious transmission and social conflict among inhabitants [11,12], and can have a negative effect on self-assessed health [13]. In addition, in recent decades, the number of studies that analyze the relationship between housing and mental health has grown considerably [14,15], with conclusions relating the issue of housing conditions to mental problems, such as increased distress, stress or difficulties in social relationships [16,17,18].

With regard to moisture problems and mold, a straight line can generally be drawn pointing to headaches, and the onset of wheezing symptoms and other respiratory problems [1,19,20,21]. Similarly, Strachan and Sanders [22] show that damp conditions in housing are a risk factor in the development of allergies; Bonnefoy et al. [23] also establish a clear relationship between these problems, and chronic anxiety and depression.

With regard to temperature, the relationship that is generally confirmed in the literature is that in cold housing where an adequate temperature cannot be maintained, respiratory and cardiovascular diseases can develop [24,25,26]. To these problems, Liddell and Guiney [27] add that low temperatures produce stressful factors, which affect people’s mental health. More recently, Llorca et al. [28] have pointed out that energy poverty has a negative impact on self-assessed health.

Furthermore, research that focuses on the effects of the neighborhood on health levels has found that living near areas with leisure and recreational activities is beneficial to a person’s well-being and mental health [29]. In addition, the existence of green spaces improves the physical and psychological health of local residents [30,31].

Conversely, a decreased feeling of neighborhood safety and increased fear of crime can result in greater psychological distress for residents [32]. High noise levels, mainly caused by locations near roads, can also have a negative effect on cardiovascular, respiratory and metabolic health [33]. These results had already been published in previous studies, which in turn highlighted the detrimental influence of pollution on health [34,35].

In addition to the aforementioned concerns, as a result of the 2008 economic crisis and its direct relationship with mortgage default problems, there was a considerable increase in research that analyzed how issues around affordability, financial burden and mortgage debt caused health problems. For example, Bentley et al. [36], Mason et al. [37] and Kavanagh et al. [38], among others, confirm that the problem of housing affordability has a negative effect on mental health. For their part, Wan and Su [8] and Corman et al. [39] affirm that housing debt and rapid price increases can result in high stress levels.

In Spain, the research that has been carried out generally establishes a negative relationship between housing deprivation and people’s health. Navarro et al. [20] determine that various forms of deprivation have a negative effect on health, while Urbanos-Garrido [40] affirms that housing deprivation explains 7.17–13.85% of the health inequality that exists between the most affluent and the most disadvantaged individuals. López del Amo González et al. [41] show that living in housing that suffers from severe material deprivation increases the probability by 70–140% that individuals will feel that they are in poor health.

Novoa et al. [42,43] use a subset of people living in substandard housing to establish that these individuals have a poor state of health in comparison with the rest of the population, and that improvements made to housing have a positive impact on self-reported health. Blázquez et al. [44] and Vásquez-Vera et al. [45] also find a negative relationship between poor housing conditions and individual health.

Even so, despite the relevant social and clinical dimensions of the problem, there is little research in Spain on how the various forms of housing deprivation affect the health of inhabitants. One reason is that much of the literature uses an indicator that groups the different forms of deprivation, making it difficult to analyze each of the factors separately. In addition, most of the existing studies on this subject have not focused on the increase in housing problems that Spain has experienced over the last decade. In other words, detailed studies have not been carried out on the physical conditions of housing and their effects on health since the onset of the 2008 economic crisis, an event that may have had a great influence. Furthermore, most of the studies carried out in Spain have generally focused on very specific social groups, so their findings cannot be generalized to include the entire population [45].

The present study aims to fill this gap by investigating the impact of housing deprivation on health, with the objective of gaining greater empirical knowledge of the phenomenon, thus allowing for the creation of better public policies. To this end, microdata is used from the National Institute of Statistics’ Living Conditions Survey (LCS hereinafter) for the period 2009–2019 [6], and several multilevel logistic regression models are presented, which make it possible to take into account the regional heterogeneity that exists in Spain. 

The results of this study suggest that there are certain structural and environmental elements related to housing that have an unquestionable effect on one’s objective state of health and the subjective perception of one’s state of health.

Those structural elements that seem to make up the multidimensional indicators of housing deprivation do not all have the same impact on the state of health and some do not seem to have any effect at all. The elements that affect objective health most notably are noise, leaks and harmful temperatures in housing. Environmental factors such as pollution, levels of vandalism or crime in the neighborhood, and the number of apartments in the building can be added to the list.

### Theoretical Framework: The Demand Model for the Good Known as “Good Health”

In order to analyze the effects produced by the various dimensional impacts that housing deprivation can have on people’s health, it is necessary to establish an analytical model that takes into consideration a variety of factors. In other words, when we consider health to be good, a health production function is identified in which the structural and environmental conditions of the dwelling are introduced as an exploratory factor [20].

To this end, we have decided to use Grossman’s model of health demand [46], which, according to Navarro et al. [20], is the most significant theoretical model for explaining people’s level of health and analyzing how they allocate their resources in the most efficient and useful way possible for the achievement of this good.

Grossman’s model affirms that health is a durable capital good possessed by each individual, whose product is the time that the individual is healthy. As such, housing deprivation becomes an element that accelerates the depreciation of health stock over time, therefore decreasing the time that the person is in good health.

In this model, consumers demand health for the following two reasons: the first is as a consumer good according to their preference functions and to avoid the disutility produced by sick days; and the second is as a form of investment in which the expected return is time available for other activities.

Furthermore, while this model establishes that health stock depreciates over time, investments can be made to improve this capital, either through direct inputs from the consumer himself/herself, such as time, or through market goods, such as diet, exercise, or housing [46]. This stock also depends on environmental variables made up mainly of human capital and various socioeconomic characteristics. All this can influence not only the productivity of the gross investment, but earlier health stock and depreciation rates.

An alternative way to analyze the effect of housing conditions on health is to adapt the health production function by incorporating variables that reflect the housing characteristics or lifestyles of individuals that could affect their health. To resolve this issue, regressors can be included in the function that provide information on individuals’ lifestyles and housing conditions, such as age, pre-existing health, educational level, or housing problems, such as the lack of some basic facilities. 

The reasoning that allows these factors to be integrated into the health production function is based on the idea that it is reasonable to think that the health of the inhabitants will be worse when the state of the dwelling does not cover the provision of functional conditions for safety, hygiene and privacy. The nature of health as a durable capital stock makes deficient housing conditions a contributing factor of its depreciation. In this sense, including housing conditions as inputs of the health production function could yield more accurate results than other standard models.

So, beyond the housing problems that are included in the health function, the regressor vector must take into account other variables that affect health stock, such as household characteristics, socioeconomic conditions, educational levels, etc. According to Navarro et al. [20], by including housing deprivation as an input of the production function for health together with these elements, more precise results can be obtained, as compared to other models.

## 2. Materials and Methods

### 2.1. Data

The sample for this study is made up of individuals over the age of 16 who responded to the Living Conditions Survey at any time from 2009 to 2019. The LCS [6] is an annual nationwide survey carried out by the INE each year, with the participation of approximately 35,000 individuals. Although the date of completion is available for each survey, the data have been pooled for our purposes.

Those observations that contained missing values (less than 2%) have been eliminated, and Cook’s distance has been used to detect possible outliers that would have an extreme influence on the model. Cook’s distance is calculated as follows:

(1) $$D_{i}=\frac{\sum_{j=1}^{n}{\left(\hat{y_{j}}-{\hat{y}}_{j\left(i\right)}\right)}^{2}{}_{\;}}{ps^{2}}$$

where $\hat{y_{j}}$ is the jth estimate, ${\hat{y}}_{j\left(i\right)}$ is this estimate without taking into account the ith observation, p is the number of terms in the model, and $s^{2}$ is the mean square error of the model. 

Unlike other methods normally used for this purpose, such as those focused on the interquartile range, this metric measures the degree of leverage of an observation, that is, the extent to which it can influence the accuracy of a regression. The higher the $D_{i}$ for observation *i*, the more it influences the coefficients for the model. As a limit for determining if an observation is too influential, a threshold of 4/n has been used, where *n* is the number of observations, and the observations that exceed this figure (approximately 5%) have been removed. Likewise, tests were carried out using other thresholds proposed by the literature, and the most conservative of these were chosen.

Ultimately, once children under 16 were removed from the database, approximately 265,000 observations remained. Even after these adjustments, the sample size continues to greatly exceed the number of individuals that make up the samples for previous studies in the field, and is considered to be a relevant contribution.

### 2.2. Method and Variables

One characteristic of the Spanish political and administrative system is its markedly decentralized nature. The regional governments in each of Spain’s 17 autonomous communities have broad powers in matters such as health and housing, so each body has the autonomy to make decisions that are significantly different from those in other regions. In addition, the socioeconomic situation in each of the regions is heterogeneous. This explains why the data for the individuals in this study have been grouped according to the autonomous community where they reside, thus creating a hierarchy between participants and their respective territories. In line with the hierarchical structure of the data that have been described, and the most recent methodological approaches in the field of housing [47,48,49,50], we have decided to use multilevel regression models to analyze housing deprivation and its effects on health.

When working with hierarchical data, the multilevel methodology provides a theoretical framework that avoids ecological and atomistic misconceptions, while helping to correctly estimate the variance that prevents incorrect interpretations [51]. This allows for the inclusion of variables related to the region in order to analyze their impact on the dependent variable, without violating the assumption of independence. In multilevel models, each “step” in the data hierarchy is represented by one level. In the present study, a two-level model has been identified, in which Level 1 shows individuals and Level 2 shows the autonomous community in which they reside.

The following two dependent variables are used: no chronic health problems and a good self-perceived state of health. For easier reading, going forward, the first variable is referred to when talking about objective health, and the second when talking about subjective health. For objective health, the LCS uses the question “Do you have any chronic illnesses or health problems?”. This variable has been recoded, taking the value 1 when the answer is “No” and the value 0 when the answer is “Yes”. For subjective health, the variable is collected using five response categories under the heading “General state of health,” ranging from a very poor state of health to a very good state of health. This variable has been dichotomized for the purposes of our analysis; the responses “very good” and “good” take the value 1, while the responses “average”, “bad” and “very bad” take the value 0.

Furthermore, we have opted to use logistic regression. The objective is to develop, for each dependent variable, three separate models that reflect different issues at the regional level, for which ecological variables are incorporated at the regional level and grouped by pairs in an effort to discover the differential incidence.

The first of the models includes regional GDP and regional GDP per capita [52], both adjusted for purchasing power standard (PPS). The second model collects decisions on health expenditures by introducing real regional public health spending both as a percentage of GDP and in per capita terms [53,54]. Finally, the third model incorporates the year-on-year change in housing prices [55] and public spending on real regional housing [56].

From there, in the development of each multilevel model, three different models are defined. The first of these, the null model, is estimated without using explanatory variables and is expressed analytically as follows:

(2) $$logit(p_{ij})=\beta_{0}+u_{ij}$$

where $\beta_{0}$ is the intercept and $u_{ij}$ is the error term of the empty model on the individual level *i* and regional level *j*. Subsequently, a second model is defined in which only the individual variables are included, as follows:

(3) $$logit(p_{ij})=\beta_{0}+\overset{H}{\underset{h=1}{\sum }}\beta_{h}X_{hij}+u_{ij}$$

where $X_{hij}$ represents the individual level variables *i*, and $\beta_{h}$ is the vector associated with their coefficients. Individual control variables include gender, age, age squared, formal studies, studies squared, the household’s ability to face unexpected expenses, employment status, housing grant received, whether the dwelling is located in a building with more than ten units, whether the individual is married, and whether the survey is administered in the midst of an economic crisis, meaning all surveys carried out before 2014.

The variables of interest represent the effects related to housing deprivation problems and their impact on health, including the following: (1) lack of light, (2) the inability to maintain an adequate temperature, (3) leaky walls or ceilings, (4) neighborhood pollution, (5) crime and (6) noise (the absence of a toilet in the housing, another indicator of housing deprivation, has been excluded, since this problem is practically non-existent in Spain, as reflected by Eurostat and the sample data (Table 1)). The use of these variables is based on the Eurostat multidimensional indicator of housing deprivation [3], which includes these physical and structural characteristics of housing. It also lists the issues related to the neighborhood, stating that the quality of a dwelling is also determined by aspects related to the physical environment in which it is located [57,58]. These variables are specified in detail in Appendix A, Table A1.

The error term in a multilevel linear model is divided into one part for each level of the model, including Level 1 (individual) and Level 2 (regional). In a two-level model, this method reveals what part of the total variability in the dependent variable is determined by Level 2 (regional, in our case) by calculating the intraclass correlation (ICC hereinafter), which in the linear model is calculated as follows:

ICC = *σ*_j_^2^/(*σ*_j_^2^ + *σ*_i_^2^) (4)

where *σ*_j_^2^ is the variance in Level 2, and *σ*_i_^2^ is the variance in Level 1. However, in a logistic model, it is not possible to clearly distinguish between the variance in both levels [59]. It is assumed that the dependent variable follows a logistic distribution in which the variance in the individual level is $\pi^{2}/3$, or 3.29, so in the logistic model, the ICC is expressed as follows [60]:

ICC = *σ*_j_^2^/(*σ*_j_^2^ + 3.29) (5)

Finally, a multilevel logistic regression model has been estimated using all the variables, as follows:

(6) $$logit(p_{ij})=\beta_{0}+\overset{H}{\underset{h=1}{\sum }}\beta_{h}X_{hij}+\overset{M}{\underset{m=1}{\sum }}\alpha_{m}X_{mj}+u_{ij}$$

In this model, the regional variables *j* has been added, which are represented by $X_{mj,}$ and the vector of coefficients associated with them is shown as $\alpha_{m}$. Both the regional variables and the continuous variables at the individual level have been standardized for analytical purposes. For the creation of the database and its analysis, Python 3.9 and the statistical package Stata 16 have been used.

## 3. Results

Table 1 shows the frequencies associated with the dichotomous variables used in the models. As for the dependent variables, 74.4% of the individuals do not have chronic health problems, and 76.9% believe they are in good health. A low percentage of the sample population has problems related to lack of light, noise, pollution, crime, leaking roofs and inadequate temperatures, but this does not pose a problem for the analysis, due to the high number of observations. There is a balanced representation of all the other control variables in the sample.

Table 2 contains descriptive statistics for the continuous variables that have been introduced in each model. Although we can observe that the housing grant variable presents values that differ markedly from the mean, these do not distort the regression results, as guaranteed by a calculation of Cook’s distance.

Conversely, the socioeconomic heterogeneity of the territories analyzed here is reflected in the statistics related to the regional variables. For example, while some territories show a 3.6 public health expenditure as a percentage of their GDP, others spent three times as much during the period in question. This same behavior is evidenced by other variables, such as GDP, GDP per capita, public health expenditures per capita, and public housing expenditures per capita.

Table 3 shows the results of the multilevel logistic regression model for the dependent variable of objective health. The individual control variables show the expected coefficients in all the models. With regard to housing deprivation, all three models show results that vary only minimally in the coefficients associated with the variables. Both noise (~0.790, *p* ≤ 0.001) and pollution (~0.880, *p* ≤ 0.05), crime (~0.767, *p* ≤ 0.001) and leaks (~0.707, *p* ≤ 0.001) lead to increases in chronic health problems. Moreover, a lack of light (~1.333, *p* ≤ 0.001) and problems maintaining adequate temperatures in housing (1.104, *p* ≤ 0.05) show a positive and statistically significant association that is present throughout the models.

In Model 1, regional GDP variables have been introduced, showing a positive association (1.228, *p* ≤ 0.001) with the dependent variable, as well as GDP per capita, which is not statistically significant. The ICC indicates that individual variability is determined on the regional level at 0.6%. The crisis variable is statistically significant and shows a positive association (1.16, *p* ≤ 0.01) with the dependent variable.

Model 2 incorporates the regional health expenditure variable both as a percentage of GDP and in per capita terms, and both are found to be statistically insignificant. Nevertheless, in this model, the ICC rises to 0.9%. Once again, the crisis variable shows a positive relationship (1.130, *p* ≤ 0.01) with the dependent variable.

In Model 3, a variable is introduced that represents the year-on-year change in housing prices, which is statistically significant in a 90% interval, and shows a negative association with the dependent variable (0.936). The public housing expenditure is also added, which is not statistically significant, neither is the crisis variable. For this model, individual variability is determined by each region at 1.5%.

Table 4 shows the results of the multilevel logistic regression model for the dependent variable of subjective health. As shown above, the control variables behave as expected. The variables related to housing deprivation show robust results that remain practically the same in the various models. Noise (~0.838, *p* ≤ 0.001), crime (~0.793, *p* ≤ 0.001), leaks (~0.659, *p* ≤ 0.001) and temperature problems (~0.961, *p* ≤ 0.1) are negatively associated with individual state of health. Conversely, a lack of light is positively associated with individual state of health (~1.228, *p* ≤ 0.001) and pollution does not have a statistically significant effect.

Model 1 results suggest that there is a positive association between regional GDP and the self-perceived good health status of individuals, while GDP per capita has no effect. Using these regional variables, individual variability is determined on the regional level at 0.9%. By comparison, Model 2 shows a positive association between subjective health and health expenditure per capita (1.029, *p* ≤ 0.05) and an ICC of 1%. The year-on-year variation in housing prices and per capita housing expenditures in Model 3 do not have a statistically significant effect on the dependent variable, but the ICC shows a notable increase to 1.3%. In this case, the effect of the crisis is shown to be robust in all three models and, unlike the pattern with chronic health problems, it has a negative (0.912, 0.903 and 0.803, respectively) and statistically significant (*p* ≤ 0.01) effect on the dependent variable.

## 4. Discussion and Conclusions

The results of the analysis make it possible to extract a series of relevant empirical considerations regarding the relationship between housing conditions and the health of Spanish citizens, both in terms of objective health and in terms of subjective or self-perceived health.

In general terms, our results confirm the initial hypothesis derived from the structure of the health production function that is taken as a theoretical reference. According to this model, poor housing conditions are assumed to determine the health status of inhabitants, once other observed and unobserved factors are controlled for.

As such, first of all, the results suggest that housing conditions can objectively influence people’s health, even after controlling for certain variables such as age, formal studies and employment status. These results can be verified using the various indicators of substandard housing considered in Models 1, 2 and 3 in Table 3, and are generally aligned with the literature, which indicates the existence of negative health effects as a result of housing conditions [9,10].

Evidently, given the difficulty of isolating and identifying the effects of poor housing conditions on health, not all substandard housing conditions considered in the models have resulted in the expected coefficients. However, likewise, these results help to correct the weights with which the various indicators of substandard housing are added for the preparation of composite indicators, such as the one prepared by Eurostat [3].

Therefore, our models indicate that noise, pollution and problems with leaks do negatively affect objective health. These results are aligned with those obtained by Recio et al. [33], with regard to noise; by Gómez-Jacinto and Hombrados-Mendieta [34], for contamination; and by Clair and Hughes [19] and Navarro et al. [20], for damp conditions.

In addition, the health of residents seems to be negatively associated with factors related to the environment around the housing, increasing the probability that health will worsen if there is crime or vandalism in the neighborhood, which aligns with the conclusions of Foster et al. [32].

Another relevant result concerning environmental variables is associated with the population density of the building in which the dwelling is located. In buildings with 10 or more units, or with a high housing density, and in which community problems may be more frequent, the models overwhelmingly suggest that the objective health of the inhabitants is worse.

Secondly, our models also make it possible to identify a series of elements in the dwelling that seem to affect both the resident’s objective health and his/her subjective perception of health Once again, these housing factors are both structural (noise and leakage problems) and environmental (particularly, neighborhood crime).

Thirdly, there are some variables that may intuitively seem to be anomalous behaviors, such as problems with maintaining an adequate temperature in the housing, but which are in fact aligned with other previous studies on this matter. As such, our models indicate that, while in objective terms, temperature problems not only do not have a negative impact on the probability of poorer health, but rather they reduce those chances; in subjective terms, the impact of temperature on health has no effect at all. These results are in line with the evidence compiled by the World Health Organization [61] regarding the connection between low indoor temperatures and adverse effects on health. The agency also found that the certainty of the evidence found is moderate, with results leading in both directions [61].

The same applies to housing that have problems with natural light, or a lack thereof. These issues do not seem to increase the probability of health issues in terms of objective or subjective health, although the coefficients are significant. This result could be explained by the fact that Spain enjoys more ambient light than other countries and so, consequently, this variable does not have the same negative impact on health as it might otherwise.

In any event, there is a noticeable lack of empirical evidence that links exposure to certain types of lighting in housing with effects on health, and most of the available evidence focuses on the effects that low exposure to sunlight during pregnancy has on fetal growth and birth, although conclusions about long-term adverse health effects have also been derived from the same study [62]. In fact, in its 2018 review, the World Health Organization hardly mentions this factor and mainly limits its comments to the role of correct lighting (whether natural or artificial) in improving accessibility and preventing domestic accidents, and not its potentially harmful effects on health [61]. Accordingly, our results underscore this same lack of negative effects.

On the other hand, it is also important to highlight that there are certain indicators of substandard housing that are not significant and as a result, it seems that they do not influence the subjective perception that inhabitants have about the causes that could explain their health problems. Such is the case with temperature and pollution problems. Previous models show that temperature does not have a negative impact on objective health, while pollution does. Taken together, these results suggest that individuals do not consider the detrimental effects that pollution has on their general state of health.

One interesting result is derived from the divergence between the impact on objective and subjective health from the density of units in a building. Here, it must be noted how, although from a subjective point of view, living in a dense community of neighbors does not seem to have any effect on health, from an objective point of view, it does. One possible hypothesis is that larger communities are potentially more conflictive, and this can have a harmful effect on health that the individual himself/herself does not fully recognize it.

Regarding the regional variables, it should be noted that most of these do not seem to affect health, except for GDP, which is positively related to both objective and subjective health. In this manner, the higher the GDP of the autonomous community, the greater the probability that the health of its inhabitants is objectively better and that, furthermore, they perceive their own health to be better. With regard to the heterogeneity of the various Spanish regions, the ICC shows individual variability depending on the region, which is similar to that obtained by other studies in this field [41].

These results are related to the new divergence reported between the coefficients of the “crisis” variable in the cases of objective and subjective health. Although during the years of the economic crisis period, there is no evidence of the impact on the objective state of health, we do observe an impact on the subjective perception of health. As such, throughout the economic crisis, the probability that a person felt that they were in poorer health increased. This result is aligned with those in the literature that confirm the increased impact of the financial burden of a mortgage or rent payment on mental health [39].

Finally, some interesting considerations can also be drawn from the control variables. There are a number of variables that have a positive impact on objective health, including educational attainment, the possibility of handling unexpected expenses, and employment status expressed in terms of job stability. In each of these cases, the better these conditions are, the better objective health individuals have on average. These results align with those in most of the literature [20].

In fact, it should be noted, especially regarding the implications that could be derived from these results in terms of public policies, that beyond the policies aimed at improving structural housing conditions and the environmental conditions of the dwelling’s surroundings, there is a positive impact from certain factors that could be modulated through policies other than those related to housing.

As such, the results indicate that both job stability and housing aid policies have a positive impact on health, both objectively and subjectively speaking. Likewise, policies for the improvement of housing conditions and housing restoration will generate positive external effects on the well-being of the population through improved health conditions.

In short, the results of the present research suggest that there are certain structural and environmental elements of housing that have a notable impact on the objective state of a person’s health and the subjective perception of that person’s state of health.

The structural elements that, in principle, make up the multidimensional indicators of housing deprivation do not all have the same impact on the state of health and some of them do not show any type of impact at all. Therefore, the elements that most affect objective health are noise, leaks and temperature problems in housing. The list continues with environmental factors such as pollution, vandalism or crime in the neighborhood, and the number of units in the building.

This study is limited in its scope. Firstly, the availability of data does not allow for analysis at the sub-regional or city level. The hierarchical structure of the data in each region is relevant because autonomous communities have a certain level of leeway with regard to key policies that could potentially affect health. Local government also has a certain capacity to make decisions that affect health, particularly on policies that generate positive health externalities, such as those of urban renewal or urban agenda, and policies that could explain the behavior of certain variables. Secondly, also concerning the high degree of decentralization of the country analyzed, the results can be extrapolated to similar systems, but may not be applicable to more centralized countries. In this respect, Spain presents a natural case that might be considered as ideal for testing the influence of regional health policy differentials; however, this, in turn, also limits the possibility of generalizing the results to other contexts.

The present analysis does not allow for the establishment of causal relationships, and it is, therefore, not possible to explain some of the relationships that are shown. The development of other studies is essential in order to help unpack the nature of these associations and implement public policies that can effectively address the problem at hand. In addition, and in light of these results, it is worth considering the need for further research on the differing factors that affect objective health and the discrepancies with those that people subjectively consider to have an effect on their health.

## Figures and Tables

**Table 1:** Frequencies associated with dichotomous variables.

Variable	0	1
No chronic health problems	25.6	74.4
Good self-perceived health	23.1	76.9
Man	51.9	48.1
Unexpected expenses	31.7	68.3
Employment status	67.9	32.1
Lack of light	96.4	3.6
Noise	85.8	14.2
Pollution	91.9	8.1
Crime	90.2	9.8
Leaking roof	86	14
No indoor toilet	99.9995	0.0005
Temperature problems	93.8	6.2
Crisis	55.8	44.2
10 or more households in building	56	44
Being married	40.9	59.1

Note: quantities are expressed as percentages.

**Table 2:** Statistical descriptions of continuous variables.

Variable	Mean	Std.	Minimum	Maximum
Age	49.988	18.637	17	86
Formal studies	2.690	1.585	0	5
Housing grant	14.792	229.362	0	14,400
GDP	102,987.7	80,844.72	8060.29	257,621.2
GDP per capita	92.655	19.305	63	136
Health expenditure as % of GDP	6.112	1.259	3.682	9.411
Health expenditure per capita	1388.65	164.441	1041.507	1876.558
Year-on-year change in housing prices	−1.407	6.886	−15.8	11.5
Housing public expenditure per capita	42.715	43.320	7	450

**Table 3:** Multilevel logistic regression models for the objective health variable.

Variable	Model 1		Model 2		Model 3	
Man	1.026		1.026		1.026	
Age	0.278	***	0.278	***	0.278	***
Age squared	1.077	**	1.077	**	1.077	**
Formal studies	1.283	***	1.283	***	1.283	***
Studies squared	0.942	***	0.942	***	0.942	***
Unexpected expenses	1.208	***	1.207	***	1.211	***
Employment status	1.739	***	1.742	***	1.746	***
Crisis	1.160	**	1.130	**	1.009	
Housing grant	1.133	***	1.134	***	1.134	***
Lack of light	1.331	***	1.335	***	1.334	***
Noise	0.790	***	0.790	***	0.791	***
Pollution	0.879	*	0.881	*	0.882	*
Crime	0.767	***	0.768	***	0.767	***
Leaking roof	0.706	***	0.707	***	0.708	***
Temperature problems	1.104	*	1.104	*	1.104	*
10 or more households in building	0.870	***	0.869	***	0.871	***
Married	0.977		0.978		0.978	
GDP	1.228	***				
GDP per capita	0.983					
Health expenditure per capita			0.992			
Health expenditure as % of GDP			0.899			
Year-on-year change in housing prices					0.936	-
Housing expenditure per capita					0.985	
Constant	3.910		3.775		3.852	
ICC	0.006		0.009		0.015	

Note: the coefficients associated with each variable are expressed as odds ratios. *p*-values: —for *p* ≤ 0.1, * for *p* ≤ 0.05, ** for *p* ≤ 0.01; *** for *p* ≤ 0.001.

**Table 4:** Multilevel logistic regression models for the subjective health variable.

Variable	Model 1		Model 2		Model 3	
Man	1.132	***	1.132	***	1.131	***
Age	0.198	***	0.198	***	0.198	***
Age squared	1.214	***	1.214	***	1.214	***
Formal studies	1.555	***	1.554	***	1.555	***
Studies squared	0.955	***	0.956	***	0.955	***
Unexpected expenses	1.664	***	1.657	***	1.665	***
Employment status	2.049	***	2.048	***	2.055	***
Crisis	0.912	**	0.903	**	0.830	**
Housing grant	1.168	***	1.168	***	1.169	***
Lack of light	1.228	***	1.229	***	1.230	***
Noise	0.838	***	0.837	***	0.838	***
Pollution	0.918		0.919		0.921	
Crime	0.793	***	0.793	***	0.794	***
Leaking roof	0.657	***	0.659	***	0.659	***
Temperature problems	0.961	-	0.962	-	0.960	-
10 or more households in building	0.987		0.984		0.988	
Married	1.032		1.033		1.033	
GDP	1.154	**				
GDP per capita	0.972					
Health expenditure per capita			1.029	*		
Health expenditure as % of GDP			0.895			
Year-on-year change in housing prices					0.957	
Housing expenditure per capita					0.980	
Constant	4.188		4.088		4.157	
ICC	0.009		0.010		0.013	

Note: the coefficients associated with each variable are expressed as odds ratios. *p*-values: —for *p* ≤ 0.1, * for *p* ≤ 0.05, ** for *p* ≤ 0.01; *** for *p* ≤ 0.001.

## Data Availability

All data are freely accessible. The processed dataset is available from the corresponding author upon reasonable request.

## Appendix A

**Table A1:** Specification of individual variables.

Variable	Description
No chronic health problems	Question: “Do you have any chronic illnesses or health problems?”. Takes the value 1 when the person responds “No” and the value 0 when the answer is “Yes”.
Good self-perceived health	Question: “Overall health”. Takes the value 1 when the individual responds “Good” or “Very good” and 0 for all other responses.
Man	The individual identifies himself as “Man” on the survey.
Formal studies	Recoded variable capturing the highest level of formal education completed by the individual. Takes the value 0 for “No studies”, the value 1 for “Primary studies or equivalent”, the value 2 for “Secondary studies or equivalent”, the value 3 for “High school of equivalent”, the value 4 for “University degree or equivalent” and the value 5 for “Postgraduate studies or equivalent”.
Unexpected expenses	Question: “Does the household have the ability to handle unexpected expenses?”. Takes the value 1 when the individual responds “Yes” and 0 when the response is “No”.
Employment status	Takes the value 1 if the individual responds that they are a full-time salaried employee.
Housing grant	Amount of money received as housing grant.
Lack of light	Question: “Is there a lack of natural light in the housing?”. Takes the value 1 when the individual responds “Yes” and the value 0 when she responds “No”.
Noise	Question: “Does the housing have problems with noise produced by neighbors or exterior surroundings (traffic, local businesses, factories, etc.)?”. Takes the value 1 when the individual responds “Yes” and the value 0 when he responds “No”.
Pollution	Question: “Does the housing have problems with pollution and dirt, or other environmental problems in the area produced by industrial activity or traffic?”. Takes the value 1 when the individual responds “Yes” and the value 0 when he responds “No”.
Crime	Question: “Are there problems with crime or vandalism in the area around your housing?”. Takes the value 1 when the individual responds “Yes” and the value 0 when she responds “No”.
Leaking roof	Question: “Does your housing have problems with leaking or dampness in the walls, floors, ceiling or foundation, or rot in the floors, doors or window frames?”. Takes the value 1 when the individual responds “Yes” and the value 0 when she responds “No”.
No indoor toilet	Question: “Does the housing have an indoor toilet and running water?”. Takes the value 1 when the response is “No” and the value 0 for all other responses.
Temperature problems	Question: “Can the household afford to keep the housing at an adequate temperature during the winter months?”. Takes the value 1 when the individual responds “No” and the value 0 when the response is “Yes”.
Crisis	Takes the value 1 when the individual took the survey before 2014 and 0 if the survey was administered during or after 2014.
10 or more households in building	Takes the value 1 if the individual resides in a building with 10 or more units and the value 0 in all other instances.
Being married	Takes the value 1 if the individual is married and 0 in all other cases.
